# Micromagnetic Simulations of Chaotic Ferromagnetic Nanofiber Networks

**DOI:** 10.3390/nano10040738

**Published:** 2020-04-12

**Authors:** Tomasz Blachowicz, Christoph Döpke, Andrea Ehrmann

**Affiliations:** 1 Institute of Physics—Center for Science and Education, Silesian University of Technology, 44-100 Gliwice, Poland; tomasz.blachowicz@polsl.pl; 2 Faculty of Engineering and Mathematics, Bielefeld University of Applied Sciences, 33619 Bielefeld, Germany; christoph.doepke@fh-bielefeld.de

**Keywords:** micromagnetic simulation, magnetic nanofiber networks, magnetization reversal, electrospinning, iron

## Abstract

Electrospinning can be used to create nanofibers with diameters of typically a few tens to a few hundred nanometers. While pure polymers are often electrospun, it is also possible to use polymer blends or to include nanoparticles. In this way, e.g., magnetic nanofiber networks can be created with a certain diameter distribution, random fiber orientations, and random crossing positions and angles. Here we present for the first time micromagnetic simulations of small parts of stochastically oriented nanofiber networks. Magnetization reversal mechanisms are investigated for different local spatial distributions; mutual influences of neighboring magnetic fibers due to dipolar interactions are depicted. This study serves as a base for the possible use of such stochastic nanofiber networks in the research area of neuro-inspired materials.

## 1. Introduction

Microscopically non-ordered systems of ferromagnetic fibers belong to the recently intensively investigated materials due to the accessible technologies of sample preparation. One of the dominating methods to produce such samples is electrospinning from liquid solutions in the presence of high electric fields. Electrospinning can be used to create nanofiber mats from diverse man-made polymers [1,2], biopolymers [3,4] or blends of polymers with non-soluble materials [5,6]. In this way, it is also possible to prepare magnetic materials by adding magnetic nanoparticles to the spinning solution [7,8].

Such magnetic nanofibers show interesting static and dynamic properties that have been investigated in theory and in experiments by many groups [9,10,11]. The influence of the bending radius of such nanofibers was found to be especially crucial [12,13,14] for the magnetization reversal dynamics. Besides basic research, nanofiber networks may be used in future in neuromorphic computing since they combine data transport paths with stochastically distributed overlapping and crossing points [13].

Until now, however, no simulations of stochastically ordered nanofiber networks or parts of them, comprising arbitrary crossing positions and angles, can be found in the scientific literature. Here we give a first overview of possible magnetization reversal processes and the influence of crossing point geometries as well as fiber cross-sections [15,16]. We discuss the impact of the numbers of crossing points and of the distance between neighboring nanofibers on coercive fields and magnetization reversal scenarios. It should be mentioned that the nanofibers under investigation here have even surfaces, as found experimentally for pure electrospinning [17,18]; opposite to fibers prepared by a new microwave plasma-based method that were recently reported, and which showed a hierarchical, complex fractal structure [19]. Rougher surfaces and possibly even fractal structures could be created by combining the electrospinning technique with additional electrospraying used in this study [20], or by carbonization or calcination of polymer/magnetic nanoparticle nanofibers, prepared by electrospinning [21,22,23]. Such technologies may lead to even more interesting magnetization reversal processes, e.g., featuring a domain wall trapping due to surface notches [24,25,26].

## 2. Simulations 

Micromagnetic simulations were carried out using two solvers. Firstly, they were based on OOMMF (Object Oriented MicroMagnetic Framework) [27] to simulate iron (Fe). Fe was chosen since our former simulation revealed interesting magnetization reversal processes in Fe nanodots [28] as well as nanofibers [13]; typical literature values were chosen as material parameters: magnetization at saturation M_S_ = 1700 × 10^3^ A/m, exchange constant A = 21 × 10^−12^ J/m, magneto-crystalline anisotropy constant K_1_ = 48 × 10^3^ J/m^3^. The Gilbert damping constant was set to α = 0.5 (equivalent to a quasi-static case).

**Figure 1:**
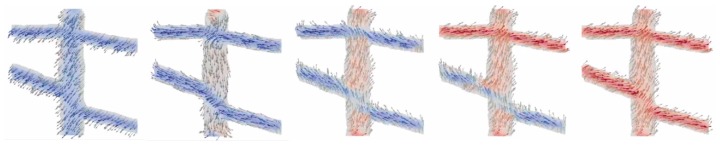
Magnetization reversal process in sample NF1 for an external magnetic field along 45° from “lower left” to “upper right” orientation. From Blachowicz et al. [29].

Two different geometries of stochastic fiber crossing points were arbitrarily chosen to mimic the SEM (scanning electron microscopy) images of real electrospun magnetic nanofiber mats and were simulated for diverse magnetic field orientations. Sample NF1 (as defined in Figure 1) corresponds to a crossing area; in sample NF2 the horizontal branches were cut so that only a single nanofiber of slightly irregular diameter remained. Besides, the thicknesses of both nanofibers were varied between round and strongly elliptical cross-sections. The latter can be produced by pressing the electrospun nanofiber mats under different conditions.

Second, the micromagnetic solver MagPar was used [30], which dynamically integrates the Landau-Lifshitz-Gilbert equation of motion and is better suited for magnetic objects in freely defined positions in a given space. Simulations were performed on sets of 10 permalloy cylinders with a length of 200 nm and a diameter of 20 nm and varying degrees of rotation freedom. For this, spherical coordinates were selected using the following expressions:

(1) $$\theta =rand\left(90^{o}-\Delta \theta ,90^{o}+\Delta \theta \right),$$

and

(2) φ=rand(0+Δφ),

where the spherical coordinates θ and φ are selected randomly from the defined ranges, and Δθ and Δφ are varied between 0° and 90° in steps of 10°. In this way, an evolution from a fully 2D set of fibers into a 3D chaotic set is performed, thus showing the influence of fiber orientation on the overall magnetization reversal process. The simulation parameters were: exchange constant *A* = 1.05 10^−11^ J/m, magnetic polarization at saturation *J*_*s*_ = 1 T, and the Gilbert damping constant *α* = 0.01.

## 3. Results and Discussion

Figure 1 depicts a magnetization reversal process of an arbitrarily chosen part of a magnetic nanofiber mat (sample NF1). It is clearly visible that due to the shape anisotropy, magnetization reversal occurs firstly in the thicker “vertical” fiber, while larger external magnetic fields are necessary to switch magnetization in the thinner “horizontal” fibers as well.

**Figure 2:**
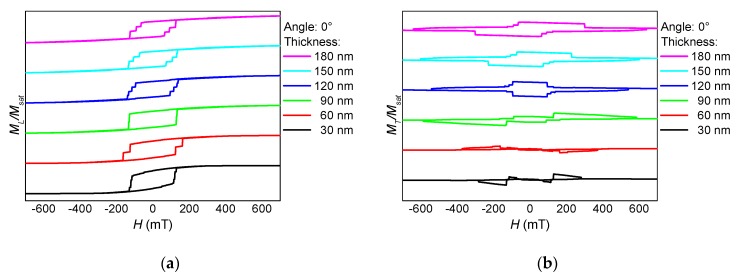
Thickness dependence of (**a**) the longitudinal and (**b**) the transverse hysteresis loops, simulated for sample NF1 under an angle of 0°.

**Figure 3:**
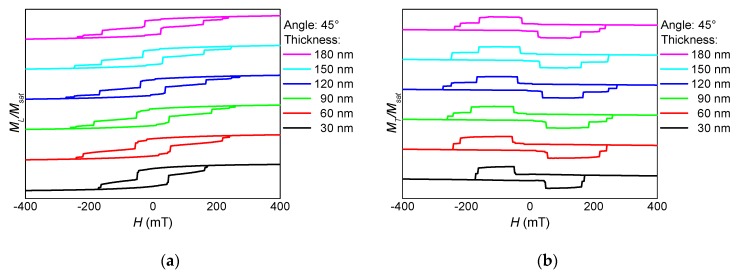
Thickness dependence of (**a**) the longitudinal and (**b**) the transverse hysteresis loops, simulated for sample NF1 under an angle of 45° (see Figure 1).

**Figure 4:**
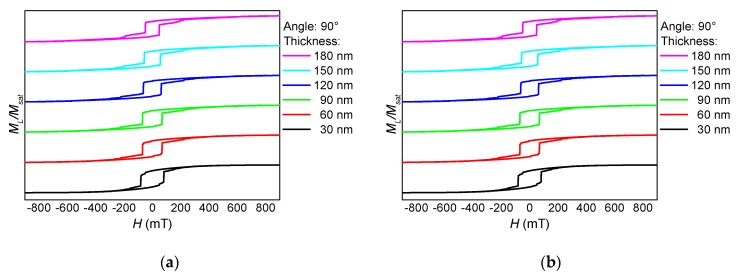
Thickness dependence of (**a**) the longitudinal and (**b**) the transverse hysteresis loops, simulated for a sample NF1 under an angle of 90°.

These separate magnetization reversal processes can also be recognized in the hysteresis loops, as depicted in Figure 2, Figure 3 and Figure 4. Here, the influence of the fiber thickness is also visible, underlining the possibility to influence the magnetic properties of such nanofiber networks by mechanical treatment after the electrospinning process. While the coercive fields are always in a similar field region, the width of the steps along the slopes of the loops differs strongly with the angular orientation and the nanofiber thickness. Such steps are correlated with irreversible magnetization reversal processes, such as nucleation and disappearance of domain walls [13,14,28], which can possibly be used for data storage in quaternary or higher-order memory devices.

**Figure 5:**
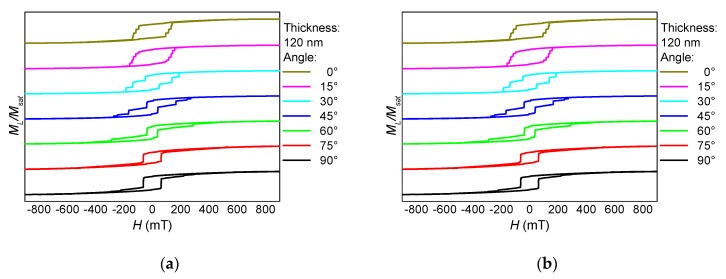
Angle dependence of (**a**) the longitudinal and (**b**) the transverse hysteresis loops, simulated for sample NF1 with a thickness of 120 nm.

The angular dependence of longitudinal and transverse hysteresis loops is depicted in Figure 5. Especially the transverse loops indicate a broad range of magnetization reversal processes, always connected with a number of steps along the hysteresis loops and thus with domain wall processes. This corresponds to the exemplary process in Figure 1, showing that the horizontal parts of this part of a nanofiber mat switch at different external magnetic fields, depending on their angular orientation and cross-section area.

It should be mentioned that all longitudinal and transverse hysteresis loops visible here and in the previous figures are not unusual for magnetic nanostructures—especially nanoparticles with crossed or bent wires or “walls”—investigated under different angles with respect to the external magnetic field [13,14,31]. The difference between the magnetic nano-structure simulated here and the common regular nanoparticles is that the “branches” and the “main cylinder” here differ in diameter, the angles between fiber parts at the intersections are arbitrary, as well as the bending radii; opposite to lithographically structured samples in which deviations from a defined geometry are undesired. This leads, e.g., to a missing fourfold symmetry (Figure 5) and thus to much more complicated angular dependencies of the longitudinal and transverse hysteresis loops as well as the coercive fields and magnetization reversal orientations (clockwise or counter-clockwise), as given by the signs of the transverse magnetization peaks.

**Figure 6:**
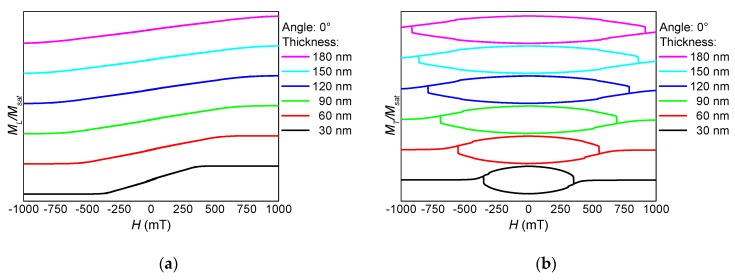
Thickness dependence of (**a**) the longitudinal and (**b**) the transverse hysteresis loops, simulated for sample NF2 under an angle of 0°.

**Figure 7:**
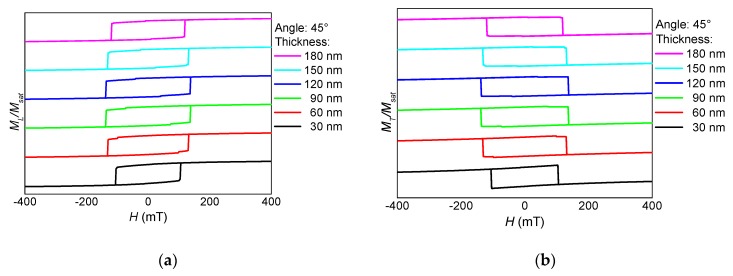
Thickness dependence of (**a**) the longitudinal and (**b**) the transverse hysteresis loops, simulated for sample NF2 under an angle of 45°.

**Figure 8:**
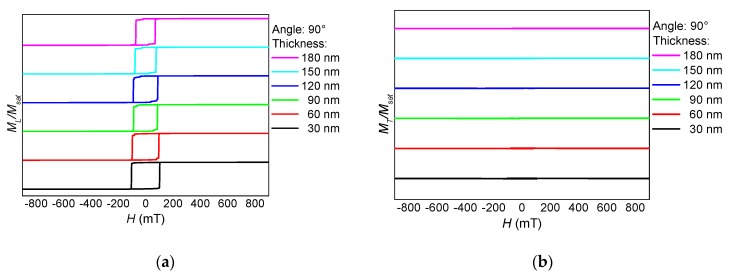
Thickness dependence of (**a**) the longitudinal and (**b**) the transverse hysteresis loops, simulated for sample NF2 under an angle of 90°.

After cutting the horizontal branches, the new sample NF2 shows a completely different behavior. As visible in Figure 6, Figure 7 and Figure 8 from the absence of steps, no domain wall processes are involved here in magnetization reversal. Instead, the 0° orientation shows the typical behavior of the magnetic field along a hard axis, with the magnetization relaxing with reduced magnetic field, as visible in the transverse o-shaped loops, and switching only at high external magnetic fields.

For 45° (Figure 7), longitudinal and transverse hysteresis loops look more like the usual Stoner-Wohlfarth-like coherent rotation of the magnetization [32], while for 90° (Figure 8), the magnetization switches without large transverse signals, i.e., without rotating out of the orientation preferred by the external magnetic field as well as the shape anisotropy.

**Figure 9:**
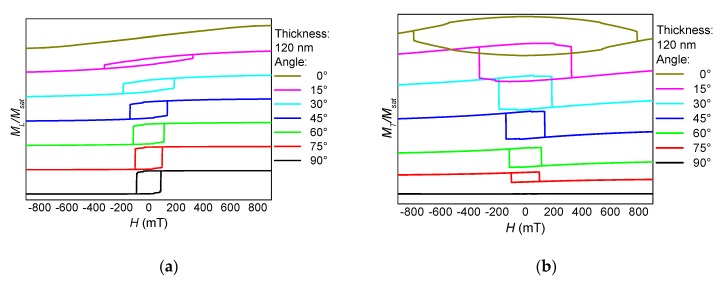
Angle dependence of (**a**) the longitudinal and (**b**) the transverse hysteresis loops, simulated for sample NF2 with a thickness of 120 nm.

The rotation of the sample NF2 at an average thickness of 120 nm again results in a strong angular dependence of the shape of longitudinal and transverse hysteresis loops as well as of the coercive fields (Figure 9). Nevertheless, these hysteresis loops look quite different from those of sample NF1, depicted in Figure 5. Here, a clear transition from a hard axis near 0° to an easy axis near 90° is visible, while this general behavior of sample NF1 was strongly superimposed by the magnetization reversal in the “horizontal branches”, making magnetization reversal processes much more interesting in the branched sample and also strongly modifying coercive fields as well as, for data storage applications more importantly, saturation fields, i.e., external magnetic fields which were sufficient to reach full magnetization reversal. 

**Figure 10:**
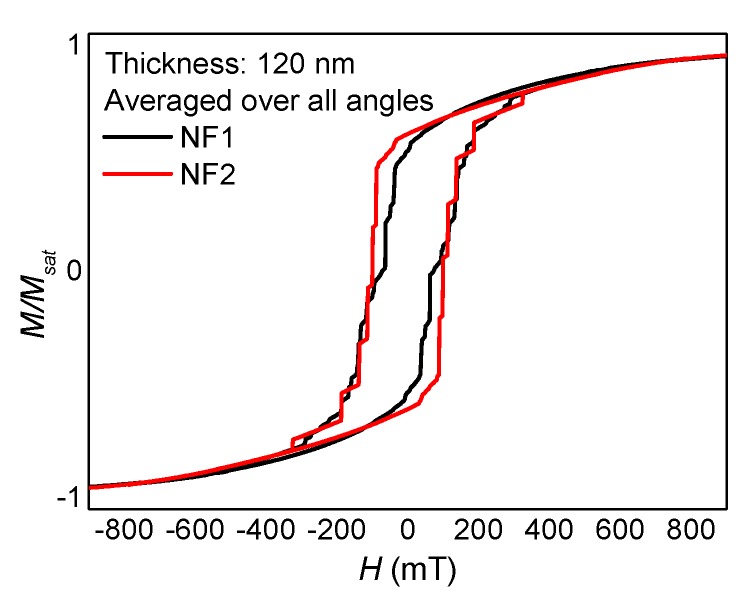
Angle dependence of the longitudinal and transverse hysteresis loops simulated for sample NF2 with a thickness of 120 nm.

This leads to the question whether the shape of a hysteresis loop, measured macroscopically by averaging over all possible angular orientations, may be used to investigate how many crossing points there are in a given electrospun sample. Figure 10 thus compares hysteresis loops averaged over the angles presented here for the samples NF1 (with branches) and NF2 (without branches). While the coercive fields are quite similar in both cases, the shapes of the loops differ nevertheless, indicating that a macroscopic measurement of the longitudinal magnetization component may indeed enable distinguishing between samples with many branches and with nearly no nodes. The numbers and dimensions of contact points can be tailored experimentally by stabilizing the nanofiber mats [33], thus this idea can be examined practically in the near future.

**Figure 11:**
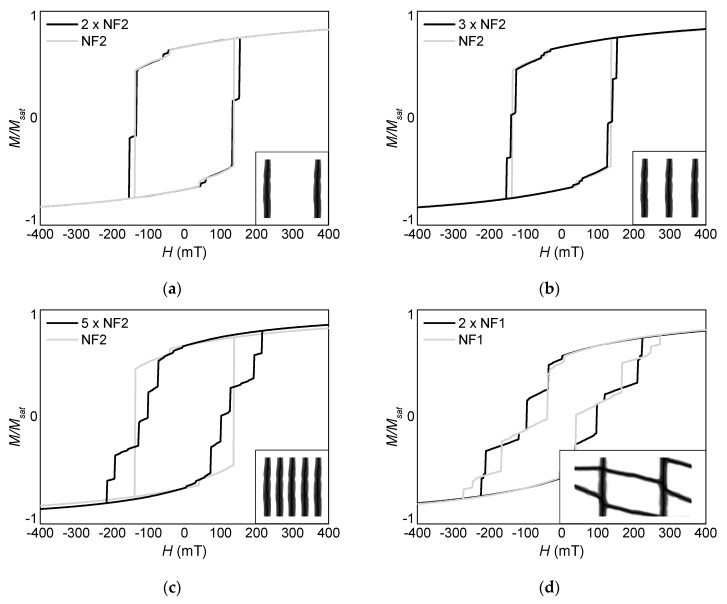
Influence of neighboring fibers in different distances on the magnetization reversal process, simulated for an angle of 45° and a thickness of 120 nm. (**a**) two neighboring fibers NF2, (**b**) three neighboring fibers NF2, (**c**) five neighboring fibers NF2, and (**d**) two adjacent fibers NF1.

Finally, the question arises how strongly neighboring fibers with or without branches (NF1 or NF2) will influence each other, depending on the distance between them. Figure 11 shows hysteresis loops for different situations, as depicted in the insets.

Adding a second nanofiber NF2 to the first one (Figure 11a), the resulting hysteresis loop is similar to the original measurement (added as light grey line). Nevertheless, there is already a step in the slope visible. Adding a third nanofiber (Figure 11b) results in an additional step, corresponding to three different external magnetic fields when the single fibers are reversed. Five nanofibers result correspondingly in a hysteresis loop with five steps, which in addition becomes much broader, i.e., magnetization reversal starts at smaller negative magnetic fields and ends at larger negative fields than in the case of the single nanofiber NF2 (Figure 11c). It should be mentioned that the order in which the fibers switch magnetization depends not only on their position, but also on the (arbitrary) orientation of the anisotropy axes in each single cell of the simulated model, corresponding to the preparation by a common sputter process. Thus, such a system is not perfectly suited for real data storage systems.

Figure 11d shows a comparison of the magnetization reversal processes of NF1 (light grey line) and a doubled version of NF1 (black line, confer inset). Here, the original branched fiber showed already four steps along the slope of the hysteresis loops, while the doubled NF1 system results in six steps of very different width and height. One can imagine that going on to extend the nanofiber network further will lead to more and more—and thus smaller and smaller—steps. This suggests changing from such macroscopic measurements and simulations to experimental and theoretical investigations of small areas, ideally of single fibers within the network, as it can be done, e.g., by magnetic force microscopy.

**Figure 12:**
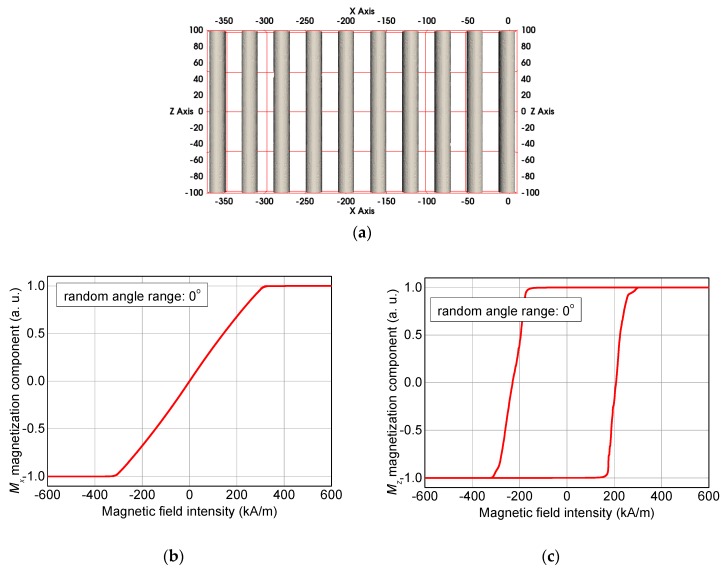
(**a**) Definition of the axes, magnetization components, (**b**) M_x_, and (**c**) M_z_ in the non-random case of 0° possible deviation of the fiber orientation from the z-orientation.

After investigating the influence of branches by OOMMF, the next simulations will show the impact of arbitrarily varying orientations of unbranched fibers, as performed by MagPar. Figure 12 defines the axes used here and gives a first idea of the magnetization components along the x-axis (M_x_) and along the z-axis (M_z_).

For the angle of 0° shown here, i.e., the non-random case of perfectly parallel fibers, the magnetization reversal for a field sweep along the x-axis shows the typical closed loop of a hard axis (Figure 12b), while sweeping the magnetization along the z-axis results in the usual shape of the hysteresis loop simulated along an easy axis.

**Figure 13:**
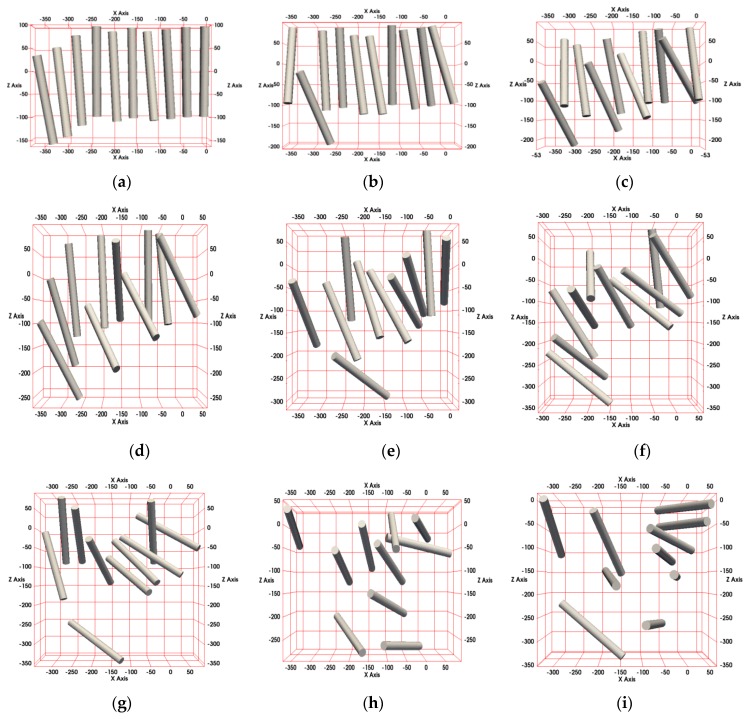
Fiber orientations used in the simulations for random angle ranges, depicted in steps of 10°: (**a**) 10°, (**b**) 20°, (**c**) 30°, (**d**) 40°, (**e**) 50°, (**f**) 60°, (**g**) 70°, (**h**) 80°, and (**i**) 90°.

The other simulated sets of fibers are depicted in Figure 13 for random angle ranges between 10° and 90°. 

**Figure 14:**
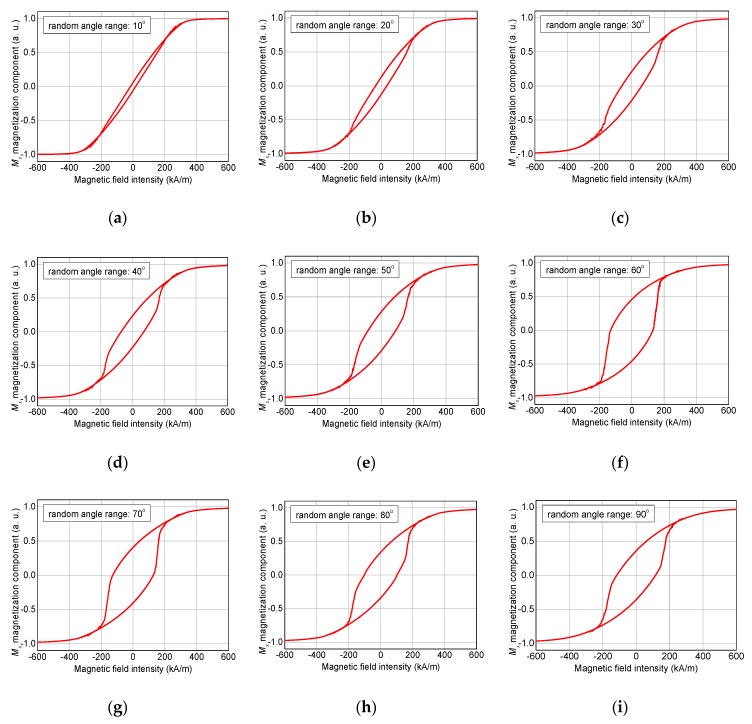
Hysteresis loops, simulated for an external magnetic field sweep along the x-axis and random angle ranges, depicted in steps of 10°: (**a**) 10°, (**b**) 20°, (**c**) 30°, (**d**) 40°, (**e**) 50°, (**f**) 60°, (**g**) 70°, (**h**) 80°, and (**i**) 90°.

**Figure 15:**
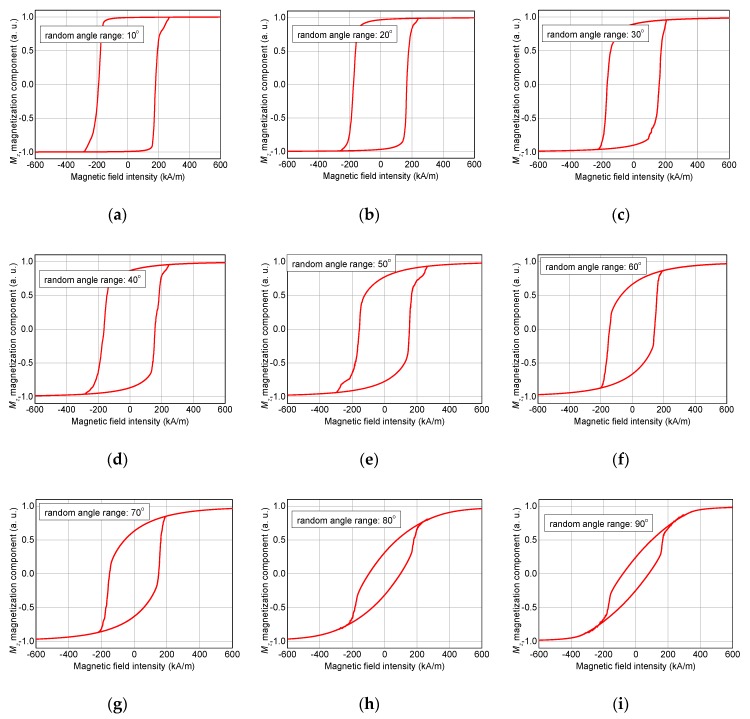
Hysteresis loops, simulated for an external magnetic field sweep along the z-axis and random angle ranges, depicted in steps of 10°: (**a**) 10°, (**b**) 20°, (**c**) 30°, (**d**) 40°, (**e**) 50°, (**f**) 60°, (**g**) 70°, (**h**) 80°, and (**i**) 90°.

Figure 14 and Figure 15 show the magnetization reversal processes for external magnetic field sweeps along the x-axis (Figure 14) or along the z-axis (Figure 15), respectively.

For the hysteresis loops simulated along the x-axis, a tendency to larger coercive fields, i.e., broader hysteresis loops for larger random angle ranges is clearly visible. Nevertheless, a hysteresis loop similar to the one simulated along the soft axis, as shown in Figure 12c, is not reached. Instead, for random angle ranges of 80° or 90°, the shapes of the hysteresis loops do not differ strongly from those simulated for random angle ranges of 50° or 60°. 

This is different for the field sweeps along the z-axis. Here, starting from typical easy axis loops for small random angle ranges, the coercive fields are still in a similar order of magnitude for simulations of random angle ranges of 40° to 60°, while the random angle ranges of 80° and 90° are similar to those simulated along the x-axis—which is obvious since for a random angle range of 90°, x- and z-axis are equivalent, and only the randomly chosen fiber orientations for the special case under simulation cause arbitrary differences.

This short overview depicts the strong influence of not only branching of fibers, but also the orientations of unbranched fibers with respect to neighboring ones and to an external magnetic field, on magnetization reversal processes and resulting hysteresis loops.

## 4. Conclusions

Magnetic iron nanofibers of varying diameters with and without branches were studied by micromagnetic simulations. In both cases, a strong dependence of longitudinal and transverse hysteresis loops on the orientation of the external magnetic field and the fiber thickness, which can be modified experimentally by mechanically pressing the nanofiber mats, was found. 

Averaging over all angles under investigation, the branched fiber and the one without branches showed slightly different longitudinal hysteresis loops. Future experimental investigations are necessary to evaluate whether such magnetic examinations reveal a macroscopic measurement for the average numbers of nodes on the nanoscale. 

The neighboring fibers were found to significantly influence the shape of hysteresis loops, offering a possible measure of the nanofiber density in a sample. This effect was even more pronounced for sets of nanofibers with angular orientations of growing randomness.

Future simulations and experiments will deal with magnetization reversal in larger-scale nanofiber networks as well as magnetization dynamics, especially the possibility of domain wall nucleation and propagation through nanofibers of different 3D orientation and along nodes between arbitrarily oriented nanofibers.
